# Efficiency in Tooth Brushing

**Published:** 1916-02

**Authors:** Carl O. Engstron

**Affiliations:** Sacramento, California


### EFFICIENCY IN TOOTH BRUSHING.

BV CARL O. ENGSTRON, D.D.S., SACRAMENTO, CALIFORNIA.

Various articles have appeared of late in dental magazines condemning the present tooth brush and its use, and simple substitutes, heretofore unknown, have been proposed. The intent of this article is to point out the two principal defects in tooth brushing as practiced today—one being the unsanitary brush itself, and the other the sweeping of food debris into protected places—and to set forth other means.

A tooth brush is defined as an instrument for cleaning teeth, in other words, for removing food accumulations, etc., from the teeth. To insure cleanliness, it must accomplish the removal of food matter from about the teeth, and the brush itself must be clean at all times. Under these conditions it may be considered ideal. Whereas the thorough cleansing with the ordinary brush, of the teeth and mouth, may not be questioned in some cases, the principles as generally applied and as described later, are very inefficient, in so far as the direct removal of food debris from the teeth and mouth and clean brush are concerned. These factors are readily understood and seem to be the contention of recent writers on the subject. Though simple may be the operation, it is none the less significant when performed at frequent periods over a length of time. Therefore, it may not be amiss to mention the principles of tooth brushing as now practiced.

In the use of the ordinary tooth brush, the brush is dipped into water with the idea of lifting the water into the mouth. As the brush is carried to the mouth, water runs onto the hand, and one movement of the bristles will cause most of the remaining water to run likewise onto the hand, leaving only moist bristles with which to clean the teeth, whereas plenty of water at this time is really needed, as much of the food debris can be removed with the water. A water syringe, as advocated by Dr. G. V. Black, is much more efficient in removing food matter than the use of these merely moist bristles. Dry as well as moist bristles often simply pass through the soft food matter. As a rule, part of the debris is swept into protected places, part is taken up by the bristles, and part is expectorated from the mouth. It will be apparent from the foregoing that some debris is left in the mouth and some on the brush in just the positions in which it should not be.

Your attention is directed to a brush which is to be connected to a water spigot, or an irrigating vessel, by means of a rubber tubing. A canal runs centrally through the handle terminating short of the further end of the brush head, and a few openings or exits for the fluid are provided between the rows of bristles.

In the use of this brush, connection is made to a hydrant or to a container of normal salt or any antiseptic solution (gravity being in this later case employed). The brush is inserted into the mouth and the water turned on. The planes of the handle are on a line with the bristles, which arrangement greatly facilitates turning the brush in the up and down movement. As the brush is moved in this manner, it will be observed that one lateral half of the bristles will pass over the gums and teeth, disturbing the food matter; then the stream of fluid issuing from the openings in the brush head will wash the debris away and out of the mouth. A space is provided for the flow of the water between the rows of bristles, and then the other lateral half of the bristles will pass over the teeth. It may be emphasized that the bristles are used merely to disturb the food matter and the running water to carry it out of the mouth. A few movements of the brush are all that is necessary, and as the stream is continuous the bristles are kept clean all the time.

This brush is self-cleansing, and when the brush is removed from the mouth it will be found to be as clean as at the beginning of the operation. No rinsing of the mouth is necessary as it practically has had a bath—an actual need in so many cases. Small bits of food, such as of meat, lodged between the teeth will be most readily felt, all mucus, etc., having been washed away. As children like to play with water, they take very eagerly and with a great deal of pleasure to this brush. The soft food matter, so often found in mouths of children, can be quickly removed. Those wearing orthodontic appliances will find that this brush will easily remove the food that accumulates around bands and wires and which induces inflammation of the soft tissues. To clean bridges in the mouth a direct flow of water, together with the action of the bristles, will render them free from food debris. In general, instead of food matter being removed by bristles, in this brush the water plays the important role in removing food accumulations from the teeth and out of the mouth, and instead of the bristles being laden with debris, they are kept clean by running water.—*Pacific Dental Gazette.*

